# Increased expression of GDF-15 may mediate ICU-acquired weakness by down-regulating muscle microRNAs

**DOI:** 10.1136/thoraxjnl-2014-206225

**Published:** 2014-12-16

**Authors:** S A A Bloch, J Y Lee, T Syburra, U Rosendahl, M J D Griffiths, P R Kemp, M I Polkey

**Affiliations:** 1 Molecular Medicine, National Heart and Lung Institute, Imperial College, London, UK; 2 National Institute for Health Research Respiratory Biomedical Research Unit, Royal Brompton & Harefield NHS Foundation Trust and Imperial College, London, UK; 3 Leukocyte Biology, National Heart and Lung Institute, Imperial College, London, UK

**Keywords:** Respiratory Muscles

## Abstract

**Rationale:**

The molecular mechanisms underlying the muscle atrophy of intensive care unit-acquired weakness (ICUAW) are poorly understood. We hypothesised that increased circulating and muscle growth and differentiation factor-15 (GDF-15) causes atrophy in ICUAW by changing expression of key microRNAs.

**Objectives:**

To investigate GDF-15 and microRNA expression in patients with ICUAW and to elucidate possible mechanisms by which they cause muscle atrophy in vivo and in vitro.

**Methods:**

In an observational study, 20 patients with ICUAW and seven elective surgical patients (controls) underwent rectus femoris muscle biopsy and blood sampling. mRNA and microRNA expression of target genes were examined in muscle specimens and GDF-15 protein concentration quantified in plasma. The effects of GDF-15 on C2C12 myotubes in vitro were examined.

**Measurements and main results:**

Compared with controls, GDF-15 protein was elevated in plasma (median 7239 vs 2454 pg/mL, p=0.001) and GDF-15 mRNA in the muscle (median twofold increase p=0.006) of patients with ICUAW. The expression of microRNAs involved in muscle homeostasis was significantly lower in the muscle of patients with ICUAW. GDF-15 treatment of C2C12 myotubes significantly elevated expression of muscle atrophy-related genes and down-regulated the expression of muscle microRNAs. miR-181a suppressed transforming growth factor-β (TGF-β) responses in C2C12 cells, suggesting increased sensitivity to TGF-β in ICUAW muscle. Consistent with this suggestion, nuclear phospho-small mothers against decapentaplegic (SMAD) 2/3 was increased in ICUAW muscle.

**Conclusions:**

GDF-15 may increase sensitivity to TGF-β signalling by suppressing the expression of muscle microRNAs, thereby promoting muscle atrophy in ICUAW. This study identifies both GDF-15 and associated microRNA as potential therapeutic targets.

**Key messages**

**What is the key question?**

- Based on our prior studies, we hypothesised that growth and differentiation factor-15 (GDF-15), a transforming growth factor-β (TGF-β) super-family member, would play a role in causing muscle atrophy in intensive care unit-acquired weakness (ICUAW).

**What is the bottom line?**

- Our data suggest that in critical illness, associated muscle wasting may, in part, be mediated by increased GDF-15 expression and consequent down-regulation of muscle-specific microRNA, which are known to control muscle homeostasis.

**Why read on?**

- This article describes a novel molecular mechanism that is potentially important in the pathogenesis of ICUAW and a novel therapeutic target.

## Introduction

Intensive care unit-acquired weakness (ICUAW) is a potentially devastating complication of critical illness. Up to 50% of patients who have been critically unwell for more than 1 week experience significant muscle wasting and weakness,^1^ associated with significant mortality, delayed weaning from mechanical ventilation, increased intensive care unit (ICU) and hospital length of stay and long-term disability.^2^ Muscle homeostasis is disturbed by an imbalance between protein synthesis and breakdown, resulting in net muscle loss.^3^ The molecular mechanisms of ICUAW are not well understood, but it is likely that the aetiology is multifactorial.^2^ ^4^ There are currently no pharmacological treatments, and few potential therapeutic targets have been identified.

Growth and differentiation factor 15 (GDF-15), a transforming growth factor-β (TGF-β) family member, is a stress-induced cytokine.^5^ GDF-15 is released from various tissues, including heart and liver, in response to inflammation, oxidative stress and hypoxia.^5^ ^6^ GDF-15 was proapoptotic in cancer cells^6^ and prevented hypertrophy of cardiac myocytes in vitro^7^ and caused cachexia in murine tumour models.^8^ We previously demonstrated that prolonged elevation in circulating GDF-15 concentrations following high-risk cardiac surgery necessitating ICU admission was associated with muscle wasting and, in vitro*,* that GDF-15 caused myotube atrophy.^9^

MicroRNAs are small non-coding RNA that regulate the translation and stability of specific mRNAs. In muscle, microRNAs modulate regeneration, differentiation and fibre type.^10^ For example, miR-1 and miR-133a fine-tune the balance between proliferation and differentiation. Inhibition of these microRNAs prevented normal proliferation and differentiation of myoblasts.^11^ MiR-181a, although not restricted to muscle, was essential for the regulation of muscle differentiation and recovery.^12^ MicroRNAs have multiple, specific mRNA targets, and altered microRNA expression has been described in muscle diseases.^10^ ^13^ MicroRNA expression can be controlled by inflammatory cytokines and, in turn, microRNAs modulate inflammatory signalling,^14^ including TGF-β pathways. For example, miR-1, miR-133a, miR-181a and miR-499 interacted with TFG-β signalling pathways in several cell lines.^15–17^ TGF-β signalling via small mothers against decapentaplegic (SMAD) protein phosphorylation is an essential pathway in muscle atrophy that can be stimulated by various ligands, including myostatin (GDF-8) and TGF-β1.^18^ In this observational study, we hypothesised that GDF-15, both muscle and from the circulation, mediates ICUAW-associated muscle atrophy through regulation of microRNA expression.

## Methods

Full method description can be found in the online repository. A brief description is given here.

### Clinical setting, patients, controls and study design

This study was carried out in a specialist cardiothoracic ICU. Patients were admitted to ICU, either following cardiothoracic surgery, from the general wards with complex and often chronic cardiorespiratory diseases, or from other centres for extracorporeal membrane oxygenation for severe acute respiratory failure. The principal inclusion criterion for this study was a diagnosis of ICUAW, made in accordance with standard diagnostic criteria;^19^ where patients were alert and cooperative, this included Medical Research Council (MRC) strength score evaluation (n=8), but in the majority their level of consciousness precluded MRC scoring. In these cases, in line with Stevens’ criteria, patients were required to have visible evidence of muscle wasting and functional evidence of muscle weakness, where other causes of muscle wasting and weakness were excluded. All adults admitted to our ICU for more than 1 week were screened. Exclusion criteria included previous neuromuscular disease, resulting in significant wasting or weakness, malignancy or contraindication to biopsy. Control participants were elective high-risk cardiothoracic surgery patients, with MRC scores 60/60 preoperatively, in whom a biopsy was taken at the start of surgery. This population was chosen to control for the complex comorbidities of patients with ICUAW. Written informed consent was obtained from study subjects or assent from the next of kin where the patient lacked capacity.

### Mid-thigh muscle layer thickness

Muscle layer thickness (MLT) of the mid-thigh was measured as previously described.^20^

### Muscle biopsy and blood processing

Rectus femoris biopsy samples were flash frozen or cork mounted and frozen. Plasma was taken at the same time as muscle biopsies and was separated from blood collected into EDTA sample tubes centrifuged at 1500 *g* (3500 rpm) for 10 min. Plasma and muscle samples were stored at −80°C. Muscle biopsy specimens were available for mRNA quantification for all seven controls and 20 patients; however, only 19 patients had sufficient RNA to allow quantification of microRNAs; microRNAs were measured in all seven controls. Adequate histology specimens were available for 4/7 controls and 7/20 patients. Plasma GDF-15 was quantified by ELISA (R&D systems, Abingdon, UK). RNA extraction and quantification, histology and immunofluorescence were carried out using validated techniques described in the online repository.

C2C12 cell culture, transfection of luciferases and overexpression of mir-181a are described in the online repository.

### Data and statistical analysis

Clinical data are described as median with IQR as the control group only consists of seven individuals. Mann–Whitney tests were used for between-group comparisons. χ^2^ tests were used to compare absolute categorical demographic data between patient and control groups. In vitro data are described as mean±SD and analysed by Student t test. Pearson's test for significant correlation was used and the resulting p values were Bonferroni corrected for multiple comparisons where appropriate. Statistical analysis and figure construction was carried out using GraphPad PRISM V.6 (GraphPad Software, California, USA).

## Results

### Patients and controls

In total, 20 of 29 patients meeting the inclusion criteria were recruited (figure E1). Seven high-risk cardiothoracic patients were recruited as controls (median EuroSCORE 9 (range 7–14)). Demographic data, comorbidities, reason for ICU admission and clinical data from ICU stay are shown in table 1. Patients and controls were well matched for age and baseline body mass index; however, the controls were all men. Owing to technical factors, including oedema, adequate ultrasound images were obtained in only five controls and 13 patients; as expected in this small sample mid-thigh MLT tended to be lower in patients than controls (1.7 (1.4–2.5) cm vs 2.9 (2.0–3.3) cm, respectively; p=0.079).

**Table 1:** Basic baseline demographic data, comorbidities and ICU data (where applicable) for patients with ICUAW and controls

	Controls (n=7)	Patients (n=20)	p Value (MW/χ^2^)
Demographics			
Age (years)	69 (63–74)	64 (51–78)	0.39
Sex (m/f)	7/0	15/5	0.14
BMI (baseline) (kg/m^2^)	24 (22–29)	27.8 (26.0–30.8)	0.21
MLT* (cm)	2.9 (2.0–3.3)	1.7 (1.4–2.5)	0.08
Comorbidities (n)			
IHD	4 of 7	8 of 20	0.42
Other cardiac disease	6 of 7	6 of 20	0.01
Respiratory	0 of 7	6 of 20	0.10
COPD	0 of 7	2 of 20	
Diabetes	1 of 7	4 of 20	0.73
Statin use	4 of 7	6 of 20	0.20
Type on cardiac surgery (n)			
CABG	1		
Valve surgery	2		
Mixed	4		
Reason for ICU admission (n)			
Postcardiac surgery		8	
Cardiac		3	
Respiratory		9	
Pneumonia		2	
COPD		2	
ARDS/ECMO		3	
Other respiratory		2	
ICU data			
MRC score at diagnosis†		36 (34–40)	
Day on ICU of biopsy (days)		20 (15–29)	
SOFA score at time of biopsy		11 (8–14)	
Total ICU LOS (days)		42 (23–51)	
Awake at biopsy (n)		6	
Fed at time of biopsy		6	
NMB (n)		11	
Corticosteroids (n)		12	
Sepsis (n)		20	
Mean CRP (mg/L)‡		136 (103–194)	
Mean pO_2_ (kPa)‡		12.68 (12.12–14.41)	
Mean CO_2_ (kPa)‡		5.7 (5.31–6.09)	
Mean pH‡		7.41 (7.40–7.44)	
Mean blood glucose (mg/dL)‡		7.6 (7.1–8.0)	
Mean cumulative insulin dose (Units)‡		456 (345–894)	
Died (1-year mortality)		8	

Data presented as median (IQR) or number (n).

*n=5 for controls and n=13 for patients.

†n=8 (six on the day of biopsy, two in the preceding 48 h).

‡During ICU stay up to the point of biopsy.

ARDS, acute respiratory distress syndrome; BMI, body mass index; CABG, coronary artery bypass graft; CRP, C-reactive protein; ECMO, extracorporeal membrane oxygenation; ICU, intensive care unit; ICUAW, intensive care unit-acquired weakness; IHD, ischaemic heart disease; LOS, length of stay; MLT, muscle layer thickness; MRC, Medical Research Council; NMB, neuromuscular blockers; SOFA, Sequential Organ Failure Assessment.

### Histology

**Figure 1:**
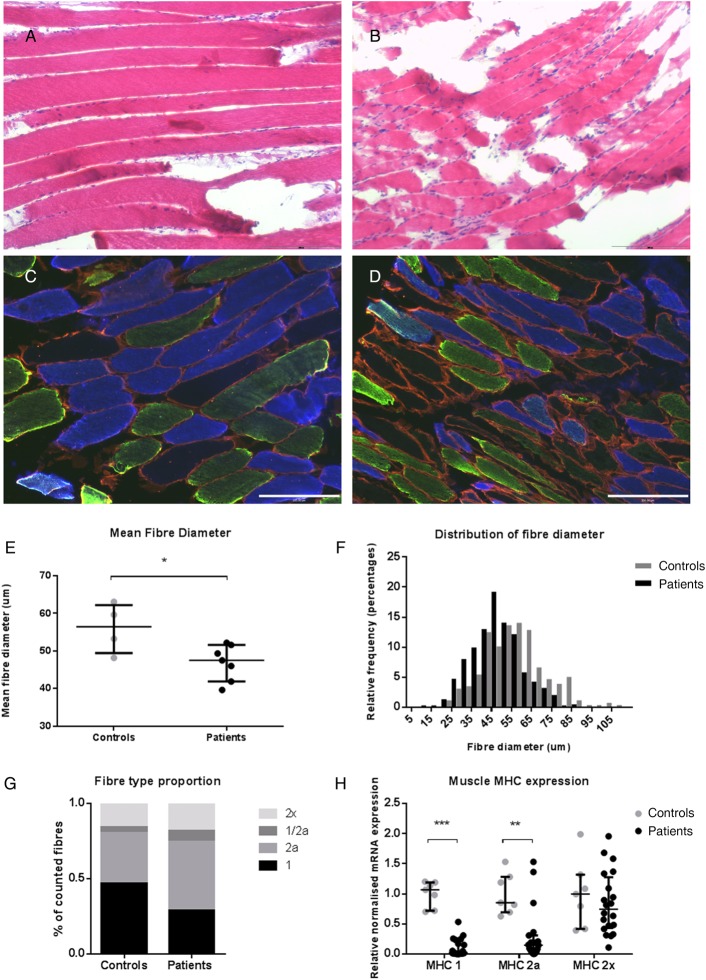
Muscle biopsy specimens from the rectus femoris of ICUAW (n=7) and controls (n=4). H&E staining of muscle biopsies from control subjects (A) and patients (B) at 10× magnification. Immunostaining of the same control (C) and patient (D) for different muscle fibre types. Samples are representative of their respective groups. Blue, MHC 1; green, MHC 2a; red, laminin: type 2× fibres do not stain and can be seen as black fibres (10× magnification). (E) Mean fibre diameter of patients and controls. (F) Percentage distribution of all fibres measured (33–107 measured per subject). (G) Mean fibre type proportion of different MHCs 1, 2a, 2x and dual staining 1/2a fibres. (H) mRNA expression for different MHCs. Controls n=7, patients n=20; data presented as median and error bars represent IQR, *p<0.05, **p<0.01, ***p<0.001 Mann–Whitney. ICUAW, intensive care unit-acquired weakness; MHC, myosin heavy chain.

Mean muscle fibre diameter for each individual was calculated from a median of 74 fibres (range 33–105 fibres/subject) depending on the size of the biopsy sample. Median mean fibre diameter was 56.4 (49.5–62.2) µm in controls and significantly lower in patients, 47.5 (41.9–51.6) µm (p=0.042). The frequency distribution of fibre size also confirmed that patients had smaller fibres as a percentage of total fibres measured (figure 1). Both MHC1 and MHC2a mRNA expression were significantly reduced in patients compared with controls (figure 1), whereas, MHC2x mRNA expression was not different. Immunofluorescent staining of specimens confirmed fibre atrophy; mean type 1 fibre diameter was 15% smaller in patients than controls (p<0.001 t test) and type 2a fibres were 24% smaller (p<0.001 t test). This analysis also showed a non-significant shift in fibre proportion towards MHC2a fibres; median proportions of MHC1 and MHC2a fibres were 44% (35–61) and 33% (28–38), respectively, in controls and 24% (21–39) and 45% (18–72) in patients (p=0.109 for type 1 fibres and 0.715 for type 2a—Mann–Whitney, figure 1).

### GDF-15 was elevated in plasma and muscle of patients

**Figure 2:**
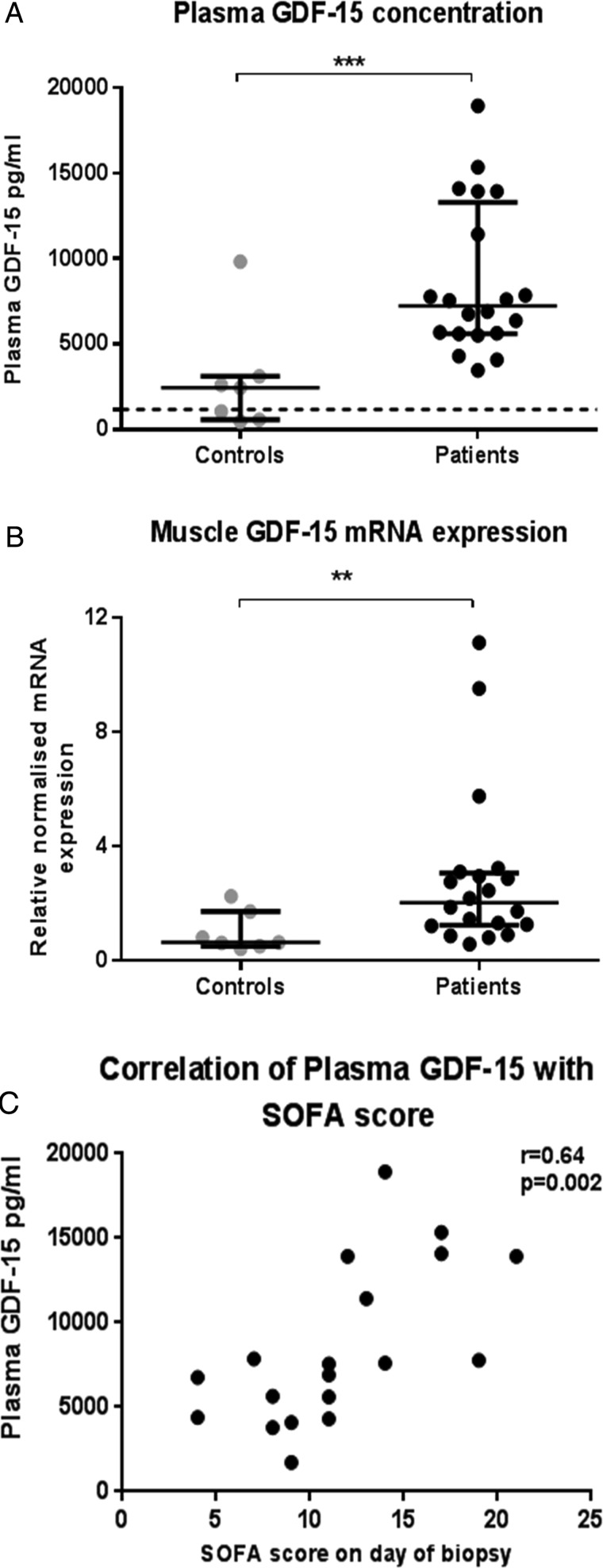
Growth and differentiation factor-15 (GDF-15) in patients with intensive care unit-acquired weakness patients (n=20) and controls (n=7) measured in plasma (A) and rectus femoris muscle biopsy mRNA expression (B). Dotted line in (A) represents 1200 pg/mL—the upper limit of normal plasma GDF-15. Data shown as median and IQR; **p<0.01, ***p<0.001—Mann–Whitney. Correlation of plasma GDF-15 with Sequential Organ Failure Assessment (SOFA) score at the time of sampling (C), r=Pearson's r value for correlation.

Median plasma GDF-15 concentration in controls was 2454 pg/mL (600–3118) and 7239 pg/mL (5613–13 295) in patients (figure 2, p=0.001). At the time of biopsy, plasma GDF-15 in the patients correlated with their Sequential Organ Failure Assessment (SOFA) score, a marker of critical illness severity^21^ (r=0.64, p=0.002).

Rectus femoris muscle mRNA expression of GDF-15 was higher in patients than controls (median 2.03-fold (1.2–3.1) higher than controls, p=0.006: figure 2).

**Figure 3:**
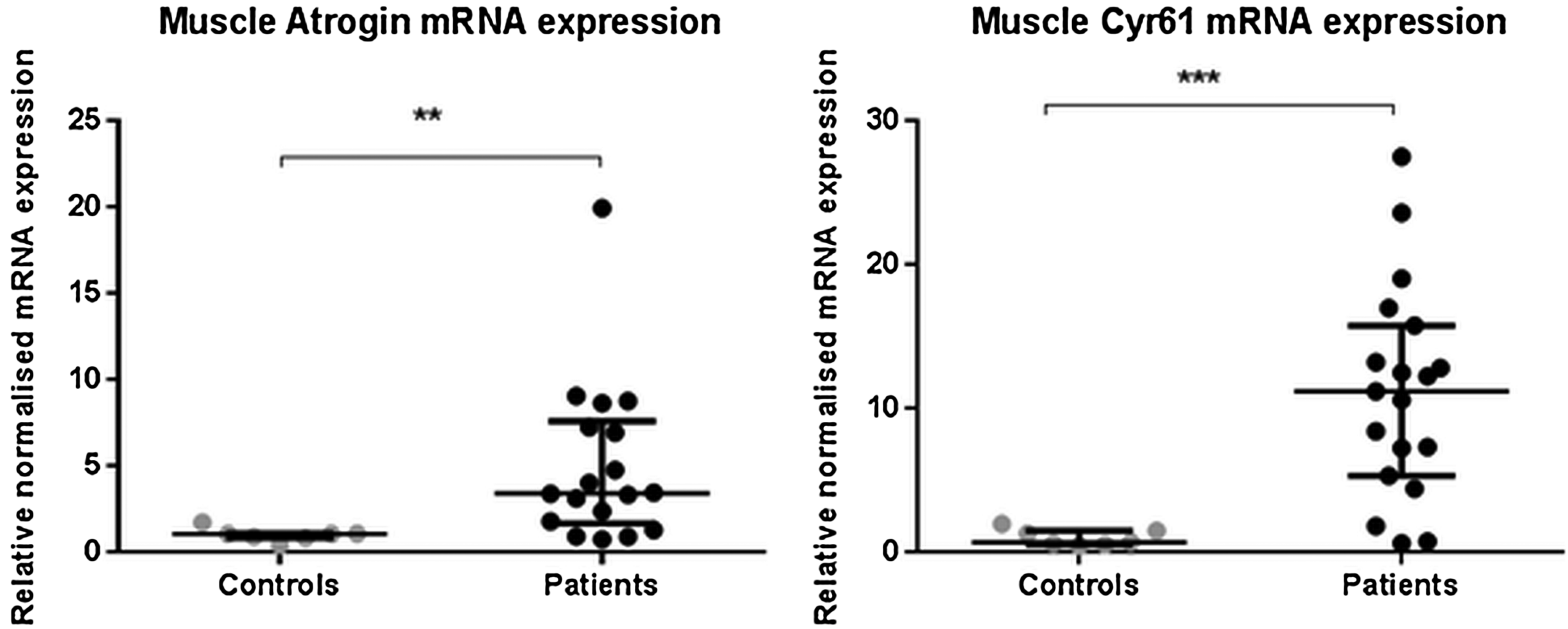
Rectus femoris muscle mRNA expression of different mRNA in patients with intensive care unit-acquired weakness (n=20) and controls (n=7) for atrogin and CYR61 (cytosine rich protein 61 (CYR61). Data presented as median and error bars represent IQR; **p<0.01, ***p<0.001 Mann–Whitney.

Neither myostatin, insulin-like growth factor-1 (IGF-1) or Muscle Ring Finger-1 (MuRF-1) mRNA expression were significantly different between patients and controls (online repository figure E2). Expression of atrogin was elevated significantly in patients (median 3.42-fold (1.7–7.9) higher than controls, p=0.003, figure 3).

### MicroRNA expression profile

**Figure 4:**
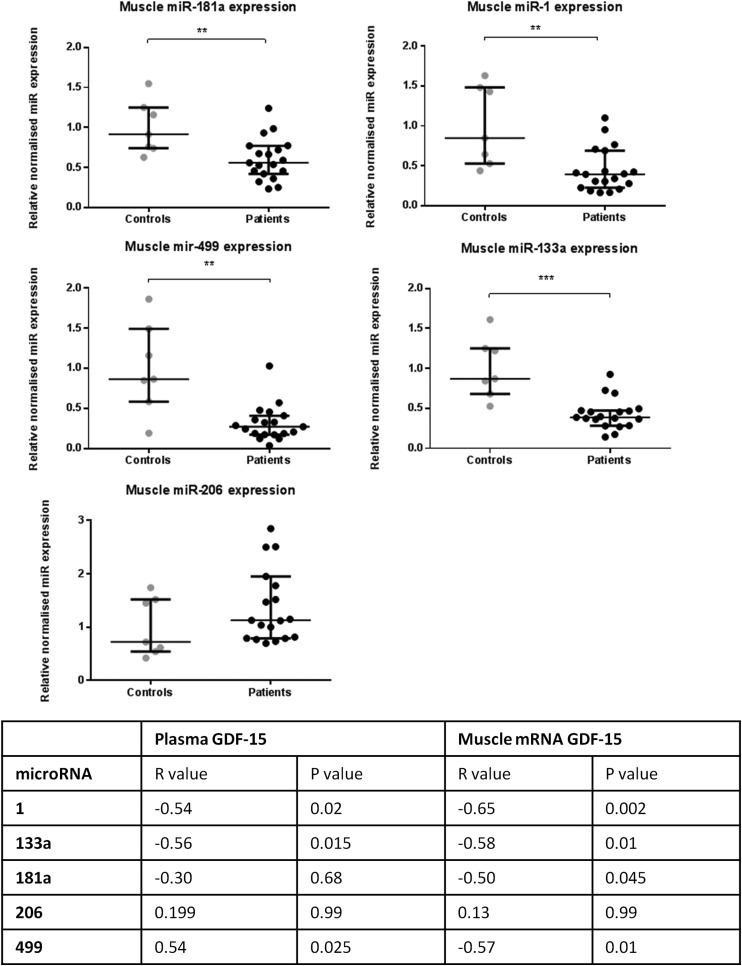
Rectus femoris muscle microRNA expression in patients with intensive care unit-acquired weakness (n=19) and controls (n=7). Table shows correlation of log (miR expression) with log (plasma growth and differentiation factor-15 (GDF-15)) and log (GDF-15 mRNA expression) Pearson’s r values and p values (Bonferroni corrected for multiple testing) are listed. Data presented as median and error bars represent IQR; **p<0.01, ***p<0.001, Mann–Whitney.

The expression of three muscle-specific microRNAs (myomiRs), miR-1, miR-133a and miR-499, were significantly reduced in the rectus femoris muscle of patients compared with controls. Median patient myomiR expression as percentage of control: miR-1 39% (22%–69%), p=0.003; miR-133a 38% (28%–47%), p<0.001; miR-49 927% (17%–41%) p=0.002. MiR-206, another myomiR, was not different between the two groups. Median miR-181a expression in patients was 56% (42%–77%) of control values, p=0.009 (figure 4). Log (GDF-15 mRNA) showed a significant negative correlation with log microRNA expression of miR-1, miR-133a, miR-181a and miR-499, but not miR-206. Log (plasma GDF-15) showed similar significant correlations with the myomiRs but not with miR-181a (figure 4).

### Increased activity of the TGF-β signalling pathway in ICUAW muscle

Cysteine-rich, angiogenic inducer-61 (CYR61) mRNA expression in ICUAW muscle, a downstream marker of TGF-β signalling,^22^ was significantly increased (median 11.18-fold (5.32–15.75) higher than controls p<0.001, figure 3).

**Figure 5:**
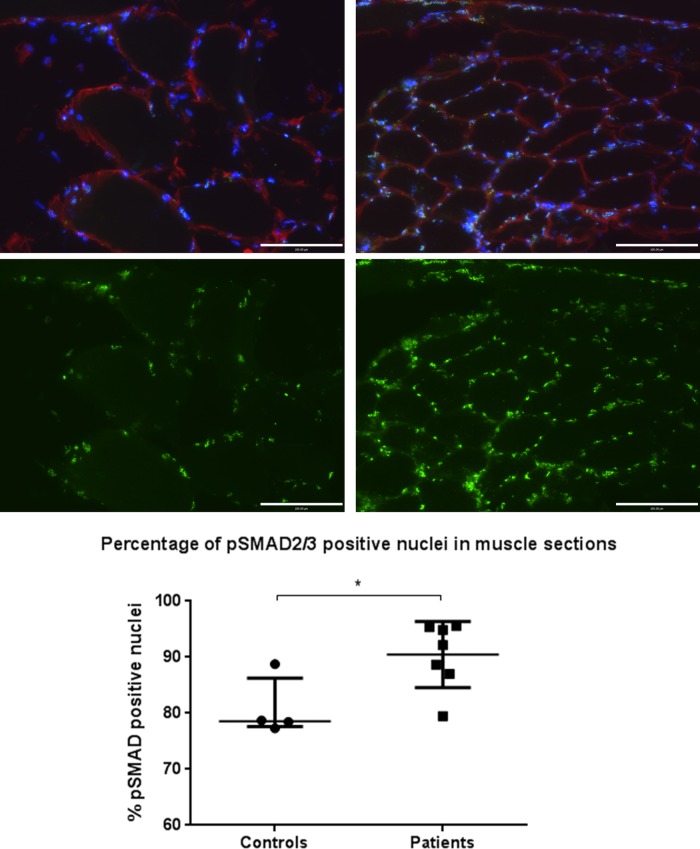
Phosphorylated small mothers against decapentaplegic 2/3 (p-SMAD 2/3) nuclear staining of muscle specimens for patients and controls. Images show control (left) and patient (right) 20× magnification muscle sections stained for p-SMAD2/3 localisation. Blue, 4′,6-diamidino-2-phenylindole nuclear; red, laminin; green, p-SMAD2/3. Lower images show p-SMAD2/3 fluorescence only of the same field of view, samples are representative of their respective groups. Graph shows percentage of pSMAD2/3-positive nuclei for controls (n=4) and patients (n=7). Data are presented as median and IQR, p=0.042, Mann–Whitney.

Binding of TGF-β family ligands (eg, TGF-β1 or myostatin) to their receptors causes phosphorylation of SMAD2/3, which in turn become localised to the nucleus and act as transcription factors for TGF-β-responsive genes.^23^ As such, nuclear positivity for phosphorylated SMAD2/3 reflects TGF-β family signalling. Immunostained muscle sections demonstrated a higher percentage of nuclei staining positive for phosphorylated SMAD2/3 in patients (median 92% (87%–95%)) vs controls (79% (78%–86%)), p=0.042 (figure 5).

### GDF-15 effects on myotubes

**Figure 6:**
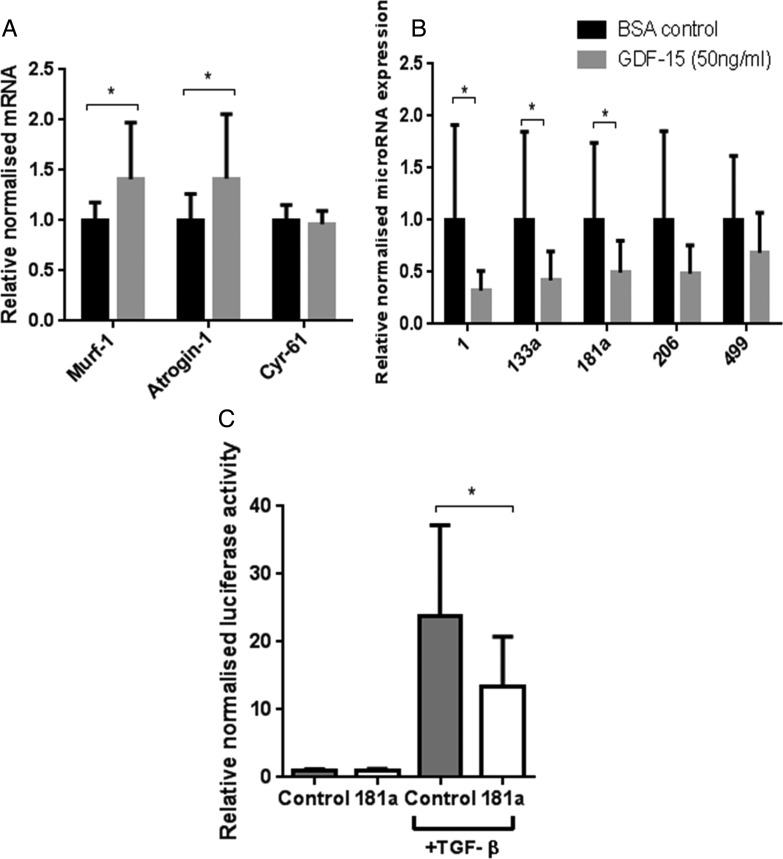
Effects of growth and differentiation factor-15 (GDF-15) or vehicle control on differentiated C2C12 myotubes. Day 8 differentiated C2C12 myotubes were treated with GDF-15 (50 ng/mL) or vehicle control (0.1% bovine serum albumin with 20 mM HCl) for 4 days, differential mRNA (A) and microRNA (B) expression was quantified (n=4 in triplicate). (C) Myoblasts were transfected with miR-181a or negative control, then CAGA-12 firefly and Renilla Luciferase plasmids. Following 6 h treatment with transforming growth factor-β (TGF-β) (2.5 ng/mL), relative luciferase activity was quantified (n=3 in triplicate). Data are normalised to their contemporary control. Data presented as mean and error bars represent SD; *p≤0.05, t test.

To determine whether GDF-15 could induce similar changes in gene expression, C2C12 myotubes were treated for 4 days with GDF-15 (50 ng/mL). MuRF-1 and atrogin mRNA expression were significantly elevated; however, CYR61 did not change (figure 6).

Consistent with the in vivo data, miR-1, miR-133a and miR-181a were reduced and miR-206 was unaltered. However, unlike in vivo, miR-499 was unchanged (figure 6).

### MiR-181a overexpression reduces TGF-β signalling

Treatment of C2C12 myoblasts with TGF-β1 (2.5 ng/mL) caused a 24-fold induction of CAGA-12-driven luciferase activity. Overexpression of miR-181a in cultured myoblasts reduced this response by 45% (figure 6).

## Discussion

**Figure 7:**
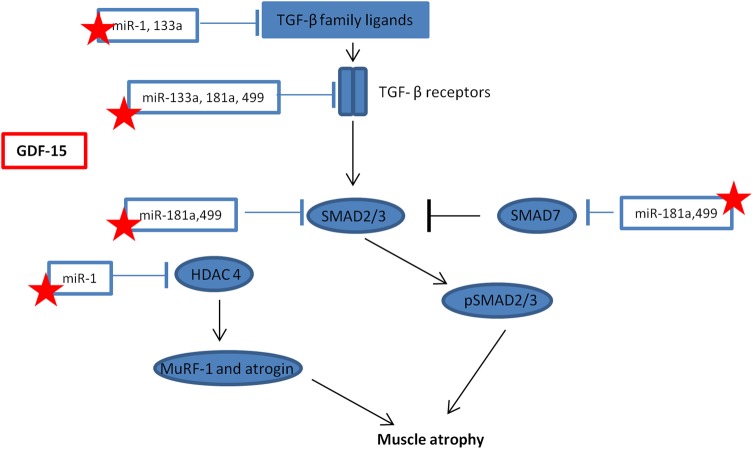
Schematic representation of the interaction between microRNAs and transforming growth factor-β (TGF-β) signalling. Stars represent those microRNA that maybe suppressed by growth and differentiation factor -15 (GDF-15) in intensive care unit-acquired weakness, resulting in a promotion of muscle atrophy. HDAC4, histone deacetylase 4; MuRF-1, Muscle Ring Finger-1; SMAD, small mothers against decapentaplegic.

The main findings of this study are that both circulating GDF-15 and muscle GDF-15 mRNA expression were elevated in patients with ICUAW compared with controls (figure 2). Moreover expression of microRNAs known to be important in muscle homeostasis and the modulation of TGF-β response were suppressed in ICUAW muscle (figure 4). Lastly GDF-15 can drive the reduction in these microRNAs in differentiated myotubes in vitro and that suppression of miR-181a may increase tissue sensitivity to TGF-β signalling (figure 6). Taken together with the evidence of increased TGF-β signalling in ICUAW muscle (figure 5), these data support the hypothesis that GDF-15 may be a mediator of acute muscle wasting in the critically ill and demonstrate that this activity may result from down-regulation of microRNAs, potentially leading to increased sensitivity to TGF-β signalling (figure 7).

The increase in circulating GDF-15 in patients with ICUAW, which is consistent with our previous study,^9^ is likely to be a result of critical illness because factors thought to contribute to ICUAW, including hyperglycaemia, inflammation and oxidative stress,^2^ ^4^ stimulate GDF-15 expression in other tissues^6^ ^24^ and, therefore, may drive the observed increase in GDF-15 expression in ICUAW muscle. The concept that circulating GDF-15 reflects illness severity is supported by our observation that patients’ plasma GDF-15 correlated with their SOFA score (figure 2) and prior observations that elevated GDF-15 in patients with acute respiratory distress syndrome predicted poor outcome.^25^

GDF-15, like other TGF-β family members, probably acts in both a paracrine and endocrine manner,^6^ and we have previously shown that GDF-15 can cause myotube atrophy in vitro;^9^ it is, therefore, feasible that elevation of both plasma and muscle GDF-15 promote ICUAW. Consistent with the concept that GDF-15 promotes myotube atrophy, GDF-15 treatment of cultured myotubes increased MuRF-1 and atrogin mRNA expression (figure 6). A direct role for GDF-15 in wasting is also consistent with the observation that GDF-15 prevented hypertrophy of cardiac myocytes;^7^ while these effects may be protective in cardiac muscle, they could promote pathological atrophy in skeletal muscle. While the concentration of GDF-15 used in vitro is higher than that measured in vivo, it is not possible to compare the relative sensitivity of the two different systems to endogenous or recombinant protein.

We showed that muscle of patients with ICUAW showed features consistent with increased TGF-β signalling, specifically increased expression of Cyr-61 and in a subgroup of patients an increased proportion of nuclei containing phosphorylated SMAD2/3 (figure 5). By contrast, in vitro studies in myoblasts or myotubes stimulated by GDF-15 did not show increased SMAD-dependent luciferase reporter gene activity (CAGA_12_-luc; data not shown) or increased Cyr-61 expression (figure 6), suggesting that these are not direct effects of GDF-15. In ICUAW muscle, we observed suppression of a number of microRNAs that inhibit various components of the TGF-β signalling pathway and, therefore, could account for the increase in Cyr-61 and nuclear p-SMAD2/3. Furthermore, GDF-15 treatment of myotubes suppressed expression of the same microRNAs, and GDF-15 mRNA expression in muscle and plasma of patients with ICUAW was inversely correlated with expression of muscle microRNAs (figure 4).

MyomiRs are essential for normal muscle homeostasis;^10^ therefore, suppression of these microRNAs by GDF-15 may contribute to muscle wasting in patients. Consistent with a protective role for these microRNAs is their ability to down-regulate components of TGF-β signalling pathways. For example, miR-1 and miR-499 (both suppressed in our patient cohort) inhibited myostatin signalling. MiR-1 increases follistatin expression by suppressing histone deacetylase 4 (HDAC4, an inhibitor of follistatin expression) and thereby inactivates circulating myostatin,^16^ miR-499 binds to the myostatin 3′-untranslated region^26^ and both are predicted to target the activin IIB receptor.^16^ ^27^ HDAC4 also suppresses the expression of muscle-specific genes^17^ and the expression of DACH2 (daschund family transcription factor 2), an indirect inhibitor of MuRF1 and atrogin expression.^28^ Thus, the reduction in miR-1 may also contribute to the reduction in MHC expression and the increase in atrogin expression we observed. MiR-133a, also suppressed in these patients,^17^ targeted TGF-β receptor type 2 and TGF-β1^29^ to down-regulate TGF-β signalling. MiR-181a, also suppressed in our patients, promoted muscle recovery following injury^12^ and decreased cell sensitivity to TGF-β signalling.^15^ Furthermore, we have demonstrated functional suppression of TGF-β signalling in response to overexpression of miR-181a in myoblasts (figure 6). Combining all of the targets (predicted by at least four databases in miRwalk,^30^ a microRNA target predicting tool) of the suppressed microRNAs in our patients with ICUAW in a pathway analysis using DAVID^31^ (a functional annotation tool for understanding the potential relationships and effects of lists of genes), highlights the TGF-β signalling pathway as one of the major pathways likely to be regulated. Indeed, the TGF-β pathway was the third most statistically significant pathway identified (p=0.005 at 2.2-fold enrichment, table E1).

We, therefore, propose that the elevation of GDF-15 in patients with ICUAW suppresses these microRNAs and thereby sensitises muscle to TGF-β signalling (figure 7). It is possible that GDF-15 sensitisation of muscle to TGF-β signalling is not unique to ICUAW. Down-regulation of muscle microRNAs is consistent with changes we have seen in COPD-associated muscle dysfunction^13^ and that others have reported in inflammatory myopathies.^14^

### Critique of the method

This study analysed a small patient population in a specialist cardiothoracic ICU. While this approach reduces the heterogeneity of participants, caution should be exercised in generalising our data to broader ICU populations, particularly given the importance of GDF-15 in cardiovascular disease.^5^ Only 8/20 patients were able to complete the MRC score for diagnosis of ICUAW; however, all patients were found to have definite evidence of visible muscle wasting and functional muscle weakness, and within the criteria set out by Stevens *et al,*^19^ the diagnosis was made by the clinical team. Unfortunately, it was not possible to assess accurately the cross-sectional area of the quadriceps muscle in these patients and controls because either the muscle was too large for a single US image or poor image quality in the patients due to oedema. Therefore, the MLT was used. Electromyography or nerve conduction studies were not undertaken in our patients. Neuropathy, usually axonal, is a recognised feature in ICUAW^2^ and we cannot exclude neuropathy as an aetiological factor in the molecular changes observed in our study.

Instead of healthy controls, our control population was deliberately selected to match the baseline medical comorbidities of this ICU's patient population, thus allowing the effect of a prolonged critical illness on muscle to be examined. We could have chosen a different control group such as patients earlier in their ICU stay prior to the clinical development of muscle wasting; however, we opted not to because molecular changes likely precede clinically overt ICUAW^3^ and such patients with similar levels of illness at an early stage of their ICU stay would already be expected to exhibit molecular changes within their muscle that cause ICUAW.

ICUAW occurs as a result of multiple pathological stimuli causing a net breakdown in muscle protein. Therefore, we might expect myostatin mRNA to be increased and IGF-1 mRNA to be reduced. However, in our patients with ICUAW, neither changed significantly (figure E2). It has recently been shown that after an initial period of reduced muscle protein synthesis a longer phase of increased catabolism predominates.^3^ Our group has previously found *increased* IGF-1 mRNA in COPD-related muscle wasting,^13^ but other groups have found *decreased* IGF-1 mRNA in human and animal muscle in response to local infection and injury.^32^ ^33^ Our patients had been critically ill for a median of 20 days, while the studies mentioned examined shorter terms. Consequently, it is important to note that different factors may contribute to ICUAW as the disease progresses, and although these patients remained critically unwell (with elevated SOFA scores), this time point may not reflect mechanisms that occurred early in the muscle insult of critical illness.

With respect to muscle protein breakdown, atrogin mRNA expression in muscle was elevated in the patient group (figure 3). Proposed mediators of ICUAW,^34^ MuRF-1 and atrogin are muscle-specific ligases that can be driven by myostatin.^18^ However, neither MuRF-1 nor myostatin mRNA was elevated in the patient's muscle (figure E2). Human studies of short term of immobilisation and acute illness^35^ have shown increased MuRF-1, but this rise was lost in the longer term,^35^ ^36^ and both human and animal data concerning myostatin are inconsistent.^34^ ^36^

A potential confounding factor in this study was the differing feeding status of patients and controls. All of our controls were fasted at the time of biopsy, for surgery. However, it was not possible to control their nutritional intake prior to surgery. By contrast, 6/20 of the patients were fed at the time of biopsy (two parenterally, four nasogastrically). Prior to enrolment in the study, 18/ 20 patients were fed continuously nasogastrically and two parenterally (because of sustained poor enteral absorption). Nutrition targets were determined by a preadmission weight-based formula at 20–25 kcal/kg/day. Feed prescription was by an ICU dietician and the patients received on average 23–25 kcal/kg/day and 1.1–1.2 g protein/kg/day. Theoretically, the metabolic status of the muscle and the balance between catabolic and anabolic signalling may differ in those who were fed and those who were not. We, therefore, reanalysed our data excluding the fed patients. No changes in the patterns of mRNA or microRNA expression described were seen—except with respect to MuRF-1. When fed patients are removed from the patient cohort, the elevation in MuRF-1 expression became statistically significant (median MuRF-1 mRNA in starved patients 1.5 (1.1–2.3) times higher than controls, p=0.011). Starvation is thought to increase catabolic drive, and it may be that MuRF-1 expression is particularly sensitive to this. Feeding effects cannot explain our in vitro observations, but it may be that GDF-15 causes muscle wasting through both direct effects on muscle and via an interaction with nutrition.

In summary, our data confirm that both circulating and muscle expression of GDF-15 are increased in patients with ICUAW and demonstrate an in vitro role for GDF-15 in muscle atrophy and in microRNA suppression. Increased GDF-15 was associated with reduced expression of muscle microRNAs, and our data raise the possibility that by doing so sensitivity to TGF-β signalling is increased. Future therapeutic options that should be explored include antibodies against GDF-15 and microRNA mimics. To date, neither of these have been trialled in humans; however, both approaches have been successfully demonstrated in murine models and one human anti-GDF-15 antibody is presently in development.

## Supplementary Material

Web supplement
