# Supplementary material for: Increased expression of GDF-15 may mediate ICU-acquired weakness by down-regulating muscle microRNAs
Source: Thorax. 2014 Dec 16;70(3):219–28. doi: 10.1136/thoraxjnl-2014-206225 (PMC4345798; doi:10.1136/thoraxjnl-2014-206225)
Supplement: Web supplement [file thoraxjnl-2014-206225-s1.pdf]

Online Repository

Increased expression of GDF-15 may mediate ICU acquired weakness by down-regulating muscle microRNAs.

Full Method description

Measurement of mid thigh muscle layer thickness

Muscle layer thickness of the mid thigh (MLT) was measured using a previously described technique [1]. Despite being validated in ICU patients [1], the MLT has potential flaws, including the impact of variable extracellular water/oedema on the measurement. However, the alternative rectus femoris cross sectional area (RF_{csa}) was not measurable in all the participants, because the whole muscle was often not visualised in a single US image. B-mode US imaging with a 10MHz 12L-RS probe was used (Logiq E, GE Healthcare, UK). The patient was positioned supine on the bed with their legs flat and leg muscles relaxed. The anterior superior iliac spine (ASIS) and the point 60% of the distance from the ASIS to the superior border of the patella were identified. The US probe was positioned perpendicular to the axis of the leg. Three separate consecutive images were taken and the MLT estimated using Image-J software (National Institutes of Health, USA). The average of these three measurements was used. Inadequate ultrasound images were defined as those where the edges of the RF muscle could not be defined well enough to calculate the MLT.

Muscle biopsy and blood sampling

Rectus femoris biopsy was taken by two methods. Open biopsy was carried out by the surgical team for the control group at the time of surgery and in those patients who were anti-coagulated or undergoing general anaesthesia for another surgical intervention, for example tracheotostomy. In the rest of the patients a closed biopsy was carried out using a Bergstrom needle as previously described [2].

Muscle samples were flash frozen in liquid nitrogen or mounted on cork in O.C.T. (VWR, UK) and frozen. Plasma was separated from blood collected into EDTA sample tubes and centrifuged at 1,500g (3500 rpm) for 10 minutes. Plasma and muscle samples were stored at

-80°C until they were processed. Where the biopsy was small, samples were prioritised for RNA and microRNA quantification, and histology was only carried out in samples that were macroscopically of good quality. Adequate histology specimens were available in 4 out of 7 controls and 7 out of 20 patients of whom 5 were male; 4 were respiratory patients, 3 were recovering from cardiac surgery, and therefore can be considered representative of the whole patient group.

Measurement of muscle fibre diameter

Mounted samples were stained with haematoxylin and eosin. Images were photographed at 10x magnification and fibre diameter measured using Image-J software. The minimal Feret's diameter, defined as the smallest distance between parallel edges of each fibre (http://www.curecmd.org/wp-content/uploads/scientists/sop/MDC1A_M.1.2.002.pdf), was measured. This distance should therefore reflect the true diameter if the fibre has been cut cross-sectionally or longitudinally.

Histology and immunofluorescence

Fresh cryosections (10 micron thick) were air dried and a ring was drawn around the section with an Immunopen (Calbiochem,UK). Sections were washed with phosphate buffered saline and 1% Tween (PBS-Tween) (Sigma, UK), then blocked with 5% fat free milk solution (Marvel, UK) in PBS-Tween. A mix of primary antibodies were prepared in PBS-Tween and incubated at 4^{0}C overnight. Sections were washed three times in PBS-Tween for 5 minutes. Secondary antibodies were prepared in PSB-Tween and the sections were incubated for 45 minutes at room temperature in the dark before being washed as above and then allowed to air dry and mounted using Fluoromount (Sigma, UK).

Primary antibodies to Myosin Heavy chains (MHC) were supplied by the Developmental Studies Hybridoma Bank, California, USA, MHC 1 (1 in 25) A4.840, MHC 2a N2.261 (1 in 50). Antibody to Laminin 4H8-2 (Abcam, UK) (1 in 500) was used for laminin staining to identify the fibre border. Alexa-Fluor® labelled secondary antibodies raised against the appropriate primary antibody species conjugated to specific fluorophores (Life Technologies, UK) were used.

The immunofluourescent signal was recorded using a Zeiss Axiovert 200 microscope and analysed with Velocity software (Perkin Elmer, Massachusetts, USA).

In a separate analysis sections were stained for nuclear localisation of p-SMAD 2/3 (primary antibody, Santa Cruz, UK) and imaged at 20x magnification. The protocol was modified, sections were fixed with 4% paraformaldehyde and then permeabilised with 0.3% Triton X100 (Sigma, UK) in PBS-Tween, to ensure nuclear permeability to the antibody, prior to blocking with 5% bovine specific albumin (BSA). Antibodies were also prepared in BSA. Nuclear localisation was achieved with 4', 6-diamino-2--phenylindole (DAPI, stock concentration at 5mg/ml diluted to 1:10000 in 1xPBS) staining (Sigma, UK).

Blood Sample analysis

Plasma level of GDF-15 was quantified using a commercially available ELISA kit (R&D systems, Abingdon, UK).

RNA extraction and quantification

RNA extraction and quantification was carried out using validated techniques. Muscle samples were homogenised using Precellys 24™ Tissue homogeniser (Stretton Scientific, UK) with 1.4mm ceramic beaded (CDK-14) tubes. RNA from muscle and *in vitro* cell culture samples was extracted using Trizol extraction (Life Technologies, UK). cDNA synthesis for mRNA was carried out using Omniscript (Qiagen, UK) and RNA was quantified using Sybr green qPCR (Quantifast® - Qiagen, UK). MicroRNAs were quantified using NCODE ™ SYBR ® green qPCR kits (Life Technologies). Full details of these methods have been previously published [3]. Primers and normalising genes used in this study can be found below.

C2C12 cell culture

C2C12 myoblasts ($1x10^5$ per well) were seeded into 6 well plates in Dulbecco's modified eagle medium (DMEM) (Life Technologies, Paisley, UK) supplemented with 10% foetal calf serum (GE Healthcare, Little Chalfont, UK) and 1% penicillin/streptomycin (Life Technologies)). The cells were grown to confluence then allowed to differentiate, forming myotubes, in differentiation medium, (DMEM supplemented with 2% horse serum (GE Healthcare), for 7 days. On the 8th day of differentiation the cells were treated with fresh

differentiation medium containing either the control vehicle (0.1% bovine serum albumin (BSA) with 20mM HCl) or 50ng/ml GDF-15 (R&D Systems) and cultured for a further 4 days.

*Transfection of luciferases and over expression of mir-181*a

C2C12 myoblasts were seeded as above. For over-expression of miR-181a, on day 1 post seeding (in a 96 well plate at 6250 cells per well) cells were transfected with miR-181a mirVANA mimic (0.5µl of a 20µM stock) or negative control (Life Technologies) using lipofectamine 2000 (Life Technologies). The following day p(CAGA)$_{12}$ luciferase and pRL-TK (Renilla luciferase) plasmids (2 µg DNA per well) were transfected with lipofectamine (Life Technologies) as previously described [4]. After 24h the cells were treated with TGF-β 2.5ng/ml (R&D Systems) or control for 6 hours. Cells were lysed in passive lysis buffer (Life Technologies, UK) and the luciferase activity measured using the dual luciferase reporting system (Promega, UK).

Primers

**mRNA sybr green primers**	
Human	
GAPDH	*F- GGTGGTCTCCTCTGACTTCAACA*<br>*R – GTTGCTGTAGCCAAATTCGTTGT*
RPLPO	*F – TCTACAACCCTGAAGTGCTTGATATC*<br>*R – GCAGACAGACACTGGCAACATT*
18s	*F- GTAACCCGTTGAACCCCATT*<br>*R- CCATCCAATCGGTAGTAGCG*
Murf-1	*F- CCTGAGAGCCATTGACTTTGG*<br>*R –CTTCCCTTCTGTGGACTCTTCCT*
Atrogin	*F- GCAGCTGAACAACATTCAGATCAC*<br>*R –CAGCCTCTGCATGATGTTCAGT*
CYR61	*F- ACTTCATGGTCCCAGTGCTC*<br>*R – TGCTGCATTTCTTGCCCTTTT*
Myostatin	*F-ACATGAACCCAGGCACTGGT*<br>*R-GGTTGTTTGAGCCAATTTTGC*
IGF-1	*F - CCACGATGCCTGTCTGAGG*<br>*R – TTTCAACAAGCCCACAGGGT*
GDF-15	*F- TGCCCGCCAGCTACAATC*<br>*R-TCTTTGGCTAACAAGTCATCATAGGT*
Mouse	
RPLPO	*F-GGACCCGAGAAGACCTCCTT*<br>*R-TGCTGCCGTTGTCAAACACC*
GAPDH	*F-ACTCCACTCCACGGCAAATTCA*

	R- CGCTCCTGGAAGATGGTGAT
Murf-1	*F-CGGGCAACGACCGAGTGCAGACGATC*<br>*R-CCAGGATGGCGTAGAGGGTGTCAAAC*
Atrogin	*F-TCAGCCTCTGCATGATGTTC*<br>*R-TGGGTGTATCGGATGGAGAC*
CYR61	*F- GGATGAATGGTGCCTTGC*<br>*R- GTCCACATCAGCCCCTTG*
**microRNA primers**	
5s	*F-ATCTCGGAAGCTAAGCA*<br>*R- GGTCTCCCATCCAAGTACT*
U6	*CTCGCTTCGGCAGCACA*
miR-1	*CCGGTGGAATGTAAAGAAGTATGTAT*
miR-133a	*GTCCCCTTCAACCAGCTG*
miR-181a	*ATTCAACGCTGTCGGTGAGT*
miR-206	*TGGAATGTAAGGAAGTGTGTGG*
miR-499	*GGCTTAAGACTTGCAGTGATGTTT*
Universal reverse	*GTACTGCGCGTGGAGAGGAAT*

Normalising genes

**Condition**	**Genes**
Human mRNAs	Geomean (RPLPO, GAPDH, 18s)
Human microRNAs	U6
Mouse mRNAs	Geomean (RPLPO, GAPDH)
Mouse microRNAs	Geomean (5s,U6)

These genes were selected on the basis of being stable between the experimental groups being compared.

Results

Table E1 to show results of DAVID analysis. Predicted targets of miRs 1,133a,181a and 499 from mirWALK were combined to generate significant analysis for significant pathways. [5-7]

Category	Term	Count	%	PValue	Bonferroni	Benjamini	FDR
KEGG_PATHWAY	hsa04320:Dorso-ventral axis formation	9	0.598007	6.64E-04	0.112042598	0.112042598	0.813624
KEGG_PATHWAY	hsa04310:Wnt signaling pathway	24	1.594684	0.003116	0.428057391	0.130362792	3.768271
**KEGG_PATHWAY**	**hsa04350:TGF-beta signaling pathway**	**16**	**1.063123**	**0.004987**	**0.591349601**	**0.13855943**	**5.96686**
BIOCARTA	h_ck1Pathway:Regulation of ck1/cdk5 by type 1 glutamate receptors	6	0.398671	0.00859	0.887250415	0.887250415	10.62069
KEGG_PATHWAY	hsa04114:Oocyte meiosis	18	1.196013	0.008977	0.800953724	0.149066923	10.50393
BIOCARTA	h_pmlPathway:Regulation of transcriptional activity by PML	6	0.398671	0.016761	0.98610804	0.88213584	19.74792
KEGG_PATHWAY	hsa04520:Adherens junction	13	0.863787	0.024415	0.988019213	0.207740145	26.22685
KEGG_PATHWAY	hsa04510:Focal adhesion	26	1.727575	0.026672	0.992086438	0.214911528	28.30056
KEGG_PATHWAY	hsa04010:MAPK signaling pathway	32	2.126246	0.034222	0.998036644	0.237386827	34.8526
KEGG_PATHWAY	hsa04062:Chemokine signaling pathway	24	1.594684	0.036555	0.998726506	0.242513535	36.76286
BIOCARTA	h_telPathway:Telomeres, Telomerase, Cellular Aging, and Immortality	6	0.398671	0.036642	0.999920888	0.957071336	38.48385
KEGG_PATHWAY	hsa04360:Axon guidance	18	1.196013	0.038208	0.999063423	0.243408008	38.08477
KEGG_PATHWAY	hsa04512:ECM-receptor interaction	13	0.863787	0.044415	0.999706113	0.268590213	42.82689

Figure E1

Consort diagram of recruitment for study

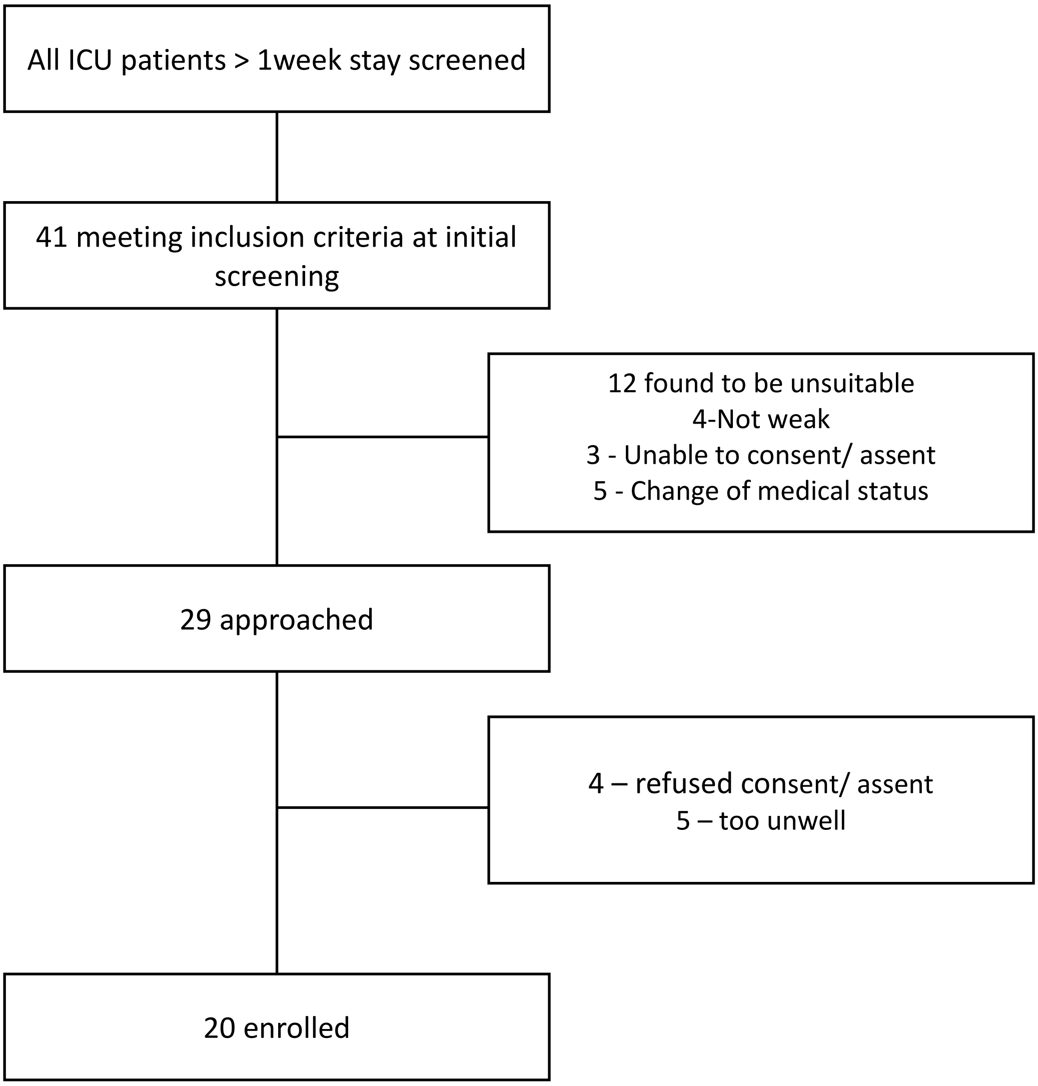

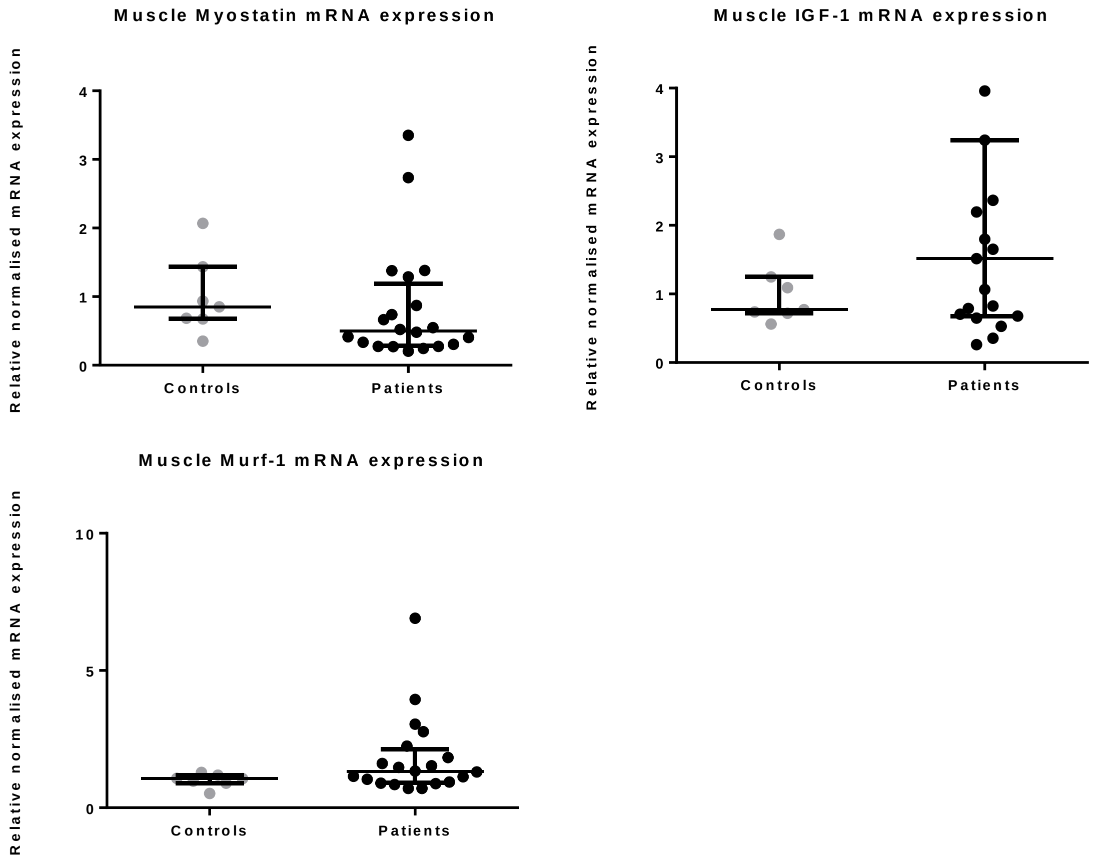

Figure E2: Rectus femoris muscle mRNA expression of different mRNA in patients with ICUAW (n=20) and controls (n=7) for Myostatin, MuRF-1 (muscle ring finger protein-1) and IGF-1 (insulin like growth factor -1). No significant differences were seen (Mann-Whitney). Data presented as median and error bars represent inter-quartile range.

References for online repository

1. Campbell, I.T., et al., *Muscle Thickness, Measured with Ultrasound, May Be an Indicator of Lean Tissue Wasting in Multiple Organ Failure in the Presence of Edema.* American Journal of Clinical Nutrition, 1995. **62**(3): p. 533-539.
2. Bergstrom, J., *Percutaneous needle biopsy of skeletal muscle in physiological and clinical research.* Scand J Clin Lab Invest, 1975. **35**(7): p. 609-16.
3. Lewis, A., et al., *Downregulation of the serum response factor/miR-1 axis in the quadriceps of patients with COPD.* Thorax, 2012. **67**(1): p. 26-34.
4. Favot, L., et al., *Cytoplasmic YY1 is associated with increased smooth muscle-specific gene expression: implications for neonatal pulmonary hypertension.* Am J Pathol, 2005. **167**(6): p. 1497-509.
5. Dweep, H., et al., *miRWalk--database: prediction of possible miRNA binding sites by "walking" the genes of three genomes.* J Biomed Inform, 2011. **44**(5): p. 839-47.
6. Huang da, W., B.T. Sherman, and R.A. Lempicki, *Systematic and integrative analysis of large gene lists using DAVID bioinformatics resources.* Nat Protoc, 2009. **4**(1): p. 44-57.
7. Huang da, W., B.T. Sherman, and R.A. Lempicki, *Bioinformatics enrichment tools: paths toward the comprehensive functional analysis of large gene lists.* Nucleic Acids Res, 2009. **37**(1): p. 1-13.
